# A strict upper bound for the partition distance and the cluster distance of phylogenetic trees for each fixed pair of topological trees

**DOI:** 10.1371/journal.pone.0204907

**Published:** 2018-09-28

**Authors:** Martin Middendorf, Nicolas Wieseke

**Affiliations:** Swarm Intelligence and Complex Systems Group, Faculty of Mathematics and Computer Science, University of Leipzig, Leipzig, Germany; Max F Perutz Laboratories GmbH, AUSTRIA

## Abstract

For each given pair of (rooted or unrooted) topological trees with the same number of leaves a strict upper bound is shown for the tree partition distance (also called symmetric difference metric and Robinson-Foulds distance)—in case of unrooted trees—and for the cluster distance (also called Robinson-Foulds distance)—in case of rooted trees—of corresponding phylogenetic trees. In particular, it is shown that there exist assignments of labels (e.g., species) to the leaves of both topological tree where each label is assigned to exactly one leaf in each tree such that: i) in the unrooted case, the tree partition distance between the corresponding phylogenetic trees equals the number of internal edges in both trees minus the number of nodes with degree 2 in both trees, ii) in the rooted case, the cluster distance between any two corresponding phylogenetic trees equals the number of internal edges in both trees minus the number of nodes with degree 2 in both trees, and iii) the values in (i) and (ii) are also the maximum values with respect to all possible assignments. The shown strict worst case bounds are needed as normalization factor to compute a normalized version of the respective tree partition metrics.

## Introduction

Tree distance measures are used in many applications to compare trees. The most widely used difference measure between (unrooted) phylogenetic trees (i.e., trees where the leaves are labelled with species) is the tree partition distance (TPD). The TPD was introduced by Bourque [1] and is also called symmetric difference metric or Robinson-Foulds metric [2] (Note, that the latter name has also been used for another tree metric that was proposed in [3]). The TDP measures the size of the symmetric difference between the two sets of bi-partitions (one set for each tree) of all labels that are obtained when for each inner edge of a tree the following is done: the edge is removed and for each of the two ermerging connected components the set of labels that are assigned to its leaves is one set of the bipartition. Several authors have studied the TPD (e.g., [4–7]).

For comparing the distances between pairs of phylogenetic trees of different sizes normalized versions of tree distance measures are used. The most often used normalization factors are strict worst case bounds. Normalized versions of the TPD have been used in several evolutionary studies (e.g., [8, 9]). To define them let *d*(*T*_1_, *T*_2_) be the TPD between two phylogenetic trees *T*_1_ and *T*_2_ which have the same number of leaves. One normalized version of the TPD (used, e.g., in [9, 10]) is then to divide the value *d*(*T*_1_, *T*_2_) by the maximum TDP for phylogenetic trees with the same number of leaves as *T*_1_ and *T*_2_. It is easy to show that maximum TPD for trees with *n* leaves is 2*n* − 6 (e.g., [6]). Instead of using a strict worst case bound as normalization factor another possibility is to use the average TPD over pairs of trees with size *n*. Specifically, in [11] the normalized TPD of trees *T*_1_ and *T*_2_ is computed as (*d*_*rand*_ − *d*(*T*_1_, *T*_2_))/*d*_*rand*_ where *d*_*rand*_ is the average TPD computed empirically over 1000 pairs of random trees of size *n* (in [11] the trees were generated with the Yule model according to a general proposal for computing normalized tree distance metrics from [12]). It should be mentioned that sometimes also TPD/2 is called normalized Robinson-Foulds distance (e.g., in [13]). Observe, that in all these definitions of normalized versions of TDP the normalization factor does not depend on the topologies of phylogenetic trees *T*_1_ and *T*_2_.

In [8] it was argued, however, that the normalization factor should consider the topologies of phylogenetic trees since not all possible phylogenetic trees are biogically relevant. Thus, a new variant of normalized TPD was proposed in [8]. In this version—denoted nTPD—value *d*(*T*_1_, *T*_2_) is divided by the worst case TPD between any two phylogenetic trees that have the same topology as *T*_1_ and *T*_2_ (but a possibly different assignment of labels to the leaves). Let *w*(*T*_1_, *T*_2_) denote this worst case value. Clearly, *w*(*T*_1_, *T*_2_) ≤ 2*n* − 6 holds. Unfortunately and to the best of our knowledge, no explicit formula for *w*(*T*_1_, *T*_2_) is known and it is not feasible for larger *n* to check all possible assignments of labels to the leaves of trees with the same topology as *T*_1_ and *T*_2_. Therefore, the computation of the nTPD in the NELSI [14] R package (function dist.topo.normalised) is (only) approximated. This is done as follows. First the TPD is computed for several pairs of phylogenetic trees that have been obtained from the two given topological trees by randomizing the assignments of labels to the leaves for one of the trees. Then the maximum TPD over all randomized pairs of phylogenetic trees is used as an approximation for the worst case TPD. In [8] the approximated nTPD was used to compare co-phylogenetic systems of different sizes and it was argued that the maximum over randomized 1000 pairs should give a good approximation. In particular, it was shown empirically for pairs of trees with sizes up to *n* = 142 that the maximum over 1000 randomizations is stable (i.e., fewer randomizations had already given the same maximum value).

In this paper we present an explicit formula to compute *w*(*T*_1_, *T*_2_). It should be mentioned that a *O*(*n*^5^) time method is given in [4] to compute for a fully resolved (i.e., each inner node has degree three) phylogenetic tree *T* with *n* leaves for all values *m* ∈ [0: 2*n* − 6] the number of phylogentic trees that have TPD *m* to *T*. By taking the maximum value *m* for which this number is not zero one obtains the worst case TDP for *T* (with respect to all other fully resolved trees with *n* leaves). But this is different from the wost case bound shown in this paper were both topological trees are fixed. Moreover, we also consider the case of trees that are not fully resolved.

In addition to the case of unrooted phylogentic trees we also consider the case of rooted phylogenetic trees. In the unrooted case the corresponding distance measure is called cluster distance (CD) or Robinson-Foulds distance for rooted trees. Recently a Robinson-Foulds metric has been proposed in [15] to compare an unrooted phylogentic tree *T*_1_ with a rooted phylogentic tree *T*_2_ when both trees have *n* leaves. The idea of this measure is to root the unrooted tree *T*_1_ optimally in the sense that the obtained rooted tree $T_{1}^{\prime }$ has a minimal CD distance to *T*_2_. Similar, as for TPD we show a strict worst case bound for CD and for the Robinson-Foulds between an unrooted phylogentic tree and a rooted phylogentic tree. Our results imply that the corresponding normalized distances can be computed efficiently.

## Basic definitions

An (unrooted) *tree* is a connected graph *T* = (*V*, *E*) with *n* = |*V*| nodes and *n* − 1 = |*E*| edges. A rooted tree is a tree which has one distiguished node with degree ≥ 2 that is called root. For a tree *T* = (*V*, *E*) a node *v* ∈ *V* is *connected* to a node *w* ∈ *V* when {*v*, *w*} ∈ *E*. A *leaf* is a node in a (rooted or unrooted) tree with degree one, a *leaf-edge* is an edge that is incident to a leaf and all other edges are *internal edges*. For an unrooted tree *T* a node that is not a leaf is an *internal node*. For a rooted tree *T* a node that is neither a leaf nor the root is an *internal node*. A *proper* (rooted or unrooted) tree is a tree *T* where each internal node has degree ≥ 3. Let T(n,m) ($T_{r}\left(n,m\right)$) be the set of all unrooted (respectively, rooted) trees with *n* leaves and *m* internal edges, *n* ≥ 3. For a proper unrooted tree T∈T(n,m) it holds that 0 ≤ *m* ≤ *n* − 3 and for a proper rooted tree $T\in T_{r}\left(n,m\right)$ it holds that 0 ≤ *m* ≤ *n* − 2.

Let *L* be a set of *n* labels. In this paper we assume w.l.o.g. *L* = {1, 2, …, *n*}. A *phylogenetic tree* on *L* is a tree *T* with *n* leaves and where each leaf is labelled with exactly one element from *L* such that for each label *l* ∈ *L* there exists a leaf with label *l*. For a phylogenetic tree the underlying tree *T* is also called the topological tree, i.e., the topological tree is the phylogenetic tree ignoring the labels of the leaves. A rooted or unrooted phylogenetic tree is *proper* if its corresponding rooted, respectively, unrooted topological tree is proper. If the context is clear notation *T* is used for a phylogenetic tree and also for its corresponding topological tree.

The removal of an edge *e* from an unrooted phylogenetic tree *T* on *L* induces a two set partition of *L*—denoted by *π*(*T*, *e*)—where each set of the partition corresponds to the labels of all nodes of one of the two connected components of *T* − *e*. Observe that for each leaf-edge *e* of *T* one set of the partition *π*(*T*, *e*) is a singleton that contains the label of the corresponding leaf. A two set partition of {1, 2, …, *n*} where one set is a singleton is called *trivial partition*. For each internal edge *e* of a proper unrooted phylogenetic tree *T* each set of the partition *π*(*T*, *e*) has at least two elements. Let *P*(*T*) be the set of all non trivial two set partitions of an unrooted phylogenetic tree *T*. For two unrooted phylogenetic trees *T*_1_ and *T*_2_ with *n* leaves the *tree partition distance* (*TPD*) between *T*_1_ and *T*_2_—denoted by *d*(*T*_1_, *T*_2_)—is the size of the symmetric difference between *P*(*T*_1_) and *P*(*T*_2_), i.e., *d*(*T*_1_, *T*_2_) = |*P*(*T*_1_) ∪ *P*(*T*_2_) − (*P*(*T*_1_) ∩ *P*(*T*_2_))|.

For each node *v* of a rooted phylogenetic tree *T* let *T*(*v*) be the subtree of *T* with root *v* and let *cl*(*T*, *v*) be the subset of *L* that contains all labels of the leaves of *T*(*v*). Set *cl*(*T*, *v*) is called the *cluster of* *v*. Observe that for each leaf *v* the cluster *cl*(*T*, *v*) is a singleton that contains the label of *v*. If *v* is the root of *T* then *cl*(*T*, *v*) = *L*. A cluster that is a singleton or equals *L* is called a *trivial cluster*. For each internal node *v* of a proper rooted phylogenetic tree *T* the cluster of *v* has at least two elements. Let *Cl*(*T*) be the set of all non trivial clusters of a rooted phylogenetic tree *T*. For two rooted phylogenetic trees *T*_1_ and *T*_2_ with *n* leaves the *cluster distance* (*CD*) between *T*_1_ and *T*_2_—denoted by *d*_*r*_(*T*_1_, *T*_2_)—is the size of the symmetric difference between *Cl*(*T*_1_) and *Cl*(*T*_2_), i.e., *d*_*r*_(*T*_1_, *T*_2_) = |*Cl*(*T*_1_) ∪ *Cl*(*T*_2_) − (*Cl*(*T*_1_) ∩ *Cl*(*T*_2_))|.

Let *T* be an unrooted phylogenetic tree. Let *E*(*T*) be the edge set of *T*. A rooting of *T* is defined by chosing an edge *e* = {*n*, *n*′} of *T* on which the root is to be placed, i.e., the edge is removed from *T*, a new node *n*″ that is the root is added to *T*, and *n*″ is connected to *n* and to *n*′. The obtained rooted tree is denoted by *T*_*e*_. According to [15] for an unrooted phylogenetic tree *T*_1_ and a rooted phylogenetic tree *T*_2_ both with *n* leaves the *unrooted cluster distance* (*urCD*) between *T*_1_ and *T*_2_—denoted by *d*_*ur*_(*T*_1_, *T*_2_)—is defined as *d*_*ur*_(*T*_1_, *T*_2_) = min_*e*∈*E*(*T*_1_)_|*Cl*(*T*_1,*e*_) ∪ *Cl*(*T*_2_) − (*Cl*(*T*_1,*e*_) ∩ *Cl*(*T*_2_))|.

## Results and discussion

For each two proper unrooted topological trees $T_{1}\in T\left(n,m_{1}\right)$ and $T_{2}\in T\left(n,m_{2}\right)$ the following upper bound on the TPD of two corresponding phylogentic trees holds: *d*(*T*_1_, *T*_2_) ≤ *m*_1_ + *m*_2_. This is clear because each internal edge can lead to at most one two set partition. If both trees are not necessarily proper and *T*_*i*_, *i* ∈ {1, 2} has *k*_*i*_ nodes with degree 2 then *d*(*T*_1_, *T*_2_) ≤ *m*_1_ − *k*_1_ + *m*_2_ − *k*_2_ holds. This result follows from the upper bound for proper trees and the fact that both edges that are incident to a node of degree 2 lead to the same two set partition of L when they are removed. Similarly, for two rooted topological trees $T_{1}\in T_{r}\left(n,m_{1}\right)$ and $T_{2}\in T_{r}\left(n,m_{2}\right)$ where *k*_*i*_ is the number of nodes of degree 2 in *T*_*i*_, *i* ∈ {1, 2} the following upper bound on the CD of two corresponding phylogentic trees holds: *d*_*r*_(*T*_1_, *T*_2_) ≤ *m*_1_ − *k*_1_ + *m*_2_ − *k*_2_. In the rest of this section we show that these upper bounds are all strict for each two unrooted (respectively rooted) topological trees *T*_1_ and *T*_2_ in the sense that there exist labelings of the leaves of *T*_1_ and *T*_2_ such that the TPD (respectively CD) of the corresponding phylogenetic trees equals the upper bound.

**Theorem 1.** *For each two proper unrooted topological trees* $T_{1}\in T\left(n,m_{1}\right)$ *and* $T_{2}\in T\left(n,m_{2}\right)$ *there exist* {1, 2,…, *n*}-*labelings of the leaves of both trees such that* *d*(*T*_1_, *T*_2_) = *m*_1_ + *m*_2_ *with* *n* ≥ 3, 0 ≤ *m*_1_ ≤ *n* − 3, 0 ≤ *m*_2_ ≤ *n* − 3.

*Proof*. For the proof we show that there exist labelings for the leaves of *T*_1_ and *T*_2_ such that for each internal edge *e*_1_ of *T*_1_ the partition *T*_1_ − *e*_1_ is not contained in *P*(*T*_2_) and for each internal edge *e*_2_ of *T*_2_ the partition *T*_2_ − *e*_2_ is not contained in *P*(*T*_1_). The proof is done by induction on *n*.

Base case *n* = 3. Since each internal node has degree three there exists exactly one internal node in each of the trees *T*_1_ and *T*_2_. Thus, *m*_1_ = *m*_2_ = 0 holds and the result follows immediatley.

For the inductive step consider two trees *T*_1_ and *T*_2_ with *n* ≥ 4 leaves and the following three cases:

1. (At least) one of the trees has only one internal node and therefore has no internal edge.
2. Both trees have (at least) one internal edge and (at least) one internal node that is connected to at least 3 leaves.
3. Both trees have (at least) one internal edge and for (at least) one of the trees each internal node is connected to at most two leaves.

Proof for case (1). W.l.o.g. let *T*_1_ be a tree that has only one internal node. Then, for any labelings of the leaves *P*(*T*_1_) = ∅ and it is clear that for each internal edge *e* of *T*_2_ the partition *π*(*T*_2_, *e*) is not in *P*(*T*_1_) and the theorem holds.

Proof for case (2). Let *u* (*x*) be an internal node in *T*_1_ (respectively *T*_2_) that is connected to at least 3 leaves. From each tree remove one of the leaves connected to *u*, respectively *x*. This does not change the number of internal edges and the resulting trees $T_{1}^{\prime }$ and $T_{2}^{\prime }$ are both proper and have *n* − 1 leaves and *m*_1_ (respectively *m*_2_) internal edges. By the induction hypothesis there exist {1, 2, …, *n* − 1}-labelings for $T_{1}^{\prime }$ and $T_{2}^{\prime }$ such that $d\left(T_{1}^{\prime },T_{2}^{\prime }\right)=m_{1}+m_{2}$. Extend these labelings to {1, 2, …, *n*}-labelings for *T*_1_ and *T*_2_ by assigning the label *n* to both removed leaves. Clearly, then *d*(*T*_1_, *T*_2_) = *m*_1_ + *m*_2_ because the number of internal edges has not changed and the bipartions of *T*_1_ (*T*_2_) are obtained from the bipartions of $T_{1}^{\prime }$ (respectively $T_{2}^{\prime }$) by adding *n* to one of the sets of each bipartition. Hence, bipartitions of $T_{1}^{\prime }$ and $T_{2}^{\prime }$ that are different are also different after adding element *n*.

It remains to prove case (3). W.l.o.g. assume that in *T*_1_ each internal node is connected to at most two leaves. Since *T*_1_ is a proper tree there exists an internal node *u* of *T*_1_ that is neighbour to exactly two leaves *u*_1_ and *u*_2_. Since *n* ≥ 4 node *u* has a neighbour *v* that is an internal node. Thus, {*u*, *v*} is an internal edge. Consider the tree $T_{1}^{\prime }$ that is obtained from *T*_1_ by removing nodes *u*_1_, *u* and edges {*u*_1_, *u*}, {*u*, *v*} and by connecting node *u*_2_ to *v*. Then $T_{1}\in T\left(n-1,m_{1}-1\right)$.

First assume that *T*_2_ has a node *x* that is connected to at least three leaves *x*_1_, *x*_2_, and *x*_3_. Construct $T_{2}^{\prime }$ by removing leaf *x*_1_. Then, $T_{2}^{\prime }\in T\left(n-1,m_{2}\right)$ holds. By the induction hypothesis there exist {1, 2, …, *n* − 1}-labelings for $T_{1}^{\prime }$ and $T_{2}^{\prime }$ such that $d\left(T_{1}^{\prime },T_{2}^{\prime }\right)=m_{1}-1+m_{2}$. Extend these labelings to {1, 2, …, *n*}-labelings for *T*_1_ and *T*_2_ by assigning leaves *u*_1_ and *x*_1_ the label *n*. Clearly, no bipartition of *P*(*T*_2_) is equal to a bipartition *π*(*T*_1_, *e*) where *e* is an internal edge with *e* ≠ {*u*, *v*}. Also, *π*(*T*_1_, {*u*, *v*}) is not in *P*(*T*_2_) because one set of *π*(*T*_1_, {*u*, *v*}) equals {*n*, *i*} for an *i* ∈ [1, *n* − 1] and all sets of bipartions in *P*(*T*_2_) that include *n* have at least three elements. Thus, *d*(*T*_1_, *T*_2_) = *m*_1_ + *m*_2_ holds.

It remains to consider the case that all nodes in *T*_2_ are connected to at most two leaves. Then there must exist an internal node *x* in *T*_2_ that is connected to two leaves *x*_1_ and *x*_2_. Clearly, *x* is connected to an internal edge {*x*, *y*}. Similar as for *T*_1_, create a phylogenetic tree $T_{2}^{\prime }$ by removing nodes *x*_1_, *x* and edges {*x*_1_, *x*}, {*x*, *y*} from *T*_2_ and by connecting *x*_2_ to *y*. By construction, $T_{2}^{\prime }$ has *n* − 1 nodes and *m*_2_ − 1 internal edges. By the induction hypothesis there exist {1, 2, …, *n* − 1}-labelings for $T_{1}^{\prime }$ and $T_{2}^{\prime }$ such that $d\left(T_{1}^{\prime },T_{2}^{\prime }\right)=m_{1}-1+m_{2}-1$. Now consider four subcases.

Case a: Both nodes *v* and *y* have at least 2 neighbouring leaves in $T_{1}^{\prime }$ respectively $T_{2}^{\prime }$. Then, we can assume w.l.o.g. that leaf *u*_2_ has label *i* in $T_{1}^{\prime }$ and leaf *x*_2_ has label *j* in $T_{2}^{\prime }$ with *j* ≠ *i* (Because, otherwise, the label of *u*_2_ can be exchanged with the label of another leaf that is connected to *v*). Now, extend the labelings to {1, 2, …, *n*}-labelings for *T*_1_ and *T*_2_ by assigning both leaves *u*_1_ and *x*_1_ label *n*. Clearly, partition *π*(*T*_1_, {*u*, *v*}) is not in *P*(*T*_2_) and partition *π*(*T*_2_, {*x*, *y*}) is not in *P*(*T*_1_). Hence, it is not hard to see that *d*(*T*_1_, *T*_2_) = *m*_1_ + *m*_2_ holds.

Case b: Node *v* has at least two neighbouring leaves (one leaf is *u*_2_ and let *v*_1_ be the other leaf) in $T_{1}^{\prime }$ and node *y* has only the neighbouring leaf (i.e., leaf *x*_2_) in $T_{2}^{\prime }$. Assume that *j* is the label of *x*_2_. Then at least one *u*_2_ and *v*_1_ has a label *i* ≠ *j*. Assume first, that node *u*_2_ has label *i* and extend the labelings of $T_{1}^{\prime }$ and $T_{2}^{\prime }$ to {1, 2, …, *n*}-labelings for *T*_1_ and *T*_2_ by assigning label *n* to leaves *u*_1_ and *x*_1_. Then the partition *π*(*T*_1_, {*u*, *v*}) is not in *P*(*T*_2_) and the partition *π*(*T*_2_, {*x*, *y*}) is not in *P*(*T*_1_). It follows easily that *d*(*T*_1_, *T*_2_) = *m*_1_ + *m*_2_ holds. It remains to consider the case that node *v*_1_ has label *i*. Then exchange the labels of *u*_2_ and *v*_1_ in $T_{1}^{\prime }$. Clearly, for this labeling $d\left(T_{1}^{\prime },T_{2}^{\prime }\right)=m_{1}-1+m_{2}-1$ holds. Now, proceed as before to show the result.

Case c: Node *v* has only one neighbouring leaf in $T_{1}^{\prime }$ and node *y* has at least 2 neighbouring leaves in $T_{2}^{\prime }$. This case is symmetric to Case (b) and the proof is analogously.

Case d: Nodes *v* and *y* have only one neighbouring leaf in $T_{1}^{\prime }$ respectively $T_{2}^{\prime }$. For the chosen {1, 2, …, *n* − 1}-labelings for $T_{1}^{\prime }$ and $T_{2}^{\prime }$ with $d\left(T_{1}^{\prime },T_{2}\right)=m_{1}-1+m_{2}-1$ let *i* and *j* be the labels of *u*_2_ respectively *x*_2_. Note, that *i* = *j* is possible. If *i* ≠ *j* extend the labelings to {1, 2, …, *n*}-labelings for *T*_1_ and *T*_2_ by assigning label *n* to leaves *u*_1_ and *x*_1_. Then, partition *π*(*T*_1_, {*u*, *v*}) is not in *P*(*T*_2_) and partition *π*(*T*_2_, {*x*, *y*}) is not in *P*(*T*_1_). Now, it is not hard to show that *d*(*T*_1_, *T*_2_) = *m*_1_ + *m*_2_ holds. It remains to consider the case *i* = *j*. Let *k* ∈ {1, 2, …, *n* − 1} be a label with *k* ≠ *i*. First, extend the labelings of $T_{1}^{\prime }$ and $T_{2}^{\prime }$ to {1, 2, …, *n*}-labelings for *T*_1_ and *T*_2_ by assigning label *n* to leaves *u*_1_ and *x*_1_. Then change the labeling for *T*_1_ by exchanging the labels of the leaves with labels *k* and *n*. Now, partition *π*(*T*_1_, {*u*, *v*}) has one set {*k*, *i*} and is therefore not in *P*(*T*_2_). Similarly, partition *π*(*T*_2_, {*x*, *y*}) has one set {*n*, *i*} and is therefore not in *P*(*T*_1_). To show that no other partion of *P*(*T*_1_) can be in *P*(*T*_2_) and vice versa, assume the contrary, i.e., assume *P*(*T*_1_) ∩ *P*(*T*_2_) ≠ ∅. Let *π*(*T*_1_, *e*_1_) = *π*(*T*_2_, *e*_2_) be a partition in *P*(*T*_1_) ∩ *P*(*T*_2_). By construction *i* and *k* must be in the same set of the partition because the corresponding leaves are connected to the same internal node in *T*_1_. Similarly, by construction *i* and *n* must be in the same set of the partition because the corresponding leaves are connected to the same internal node in *T*_2_. Hence *e*_1_ ≠ {*u*, *v*} and *e*_2_ ≠ {*x*, *y*}. Altogether it follows that $\pi \left(T_{1}^{\prime },e_{1}\right)=\pi \left(T_{2}^{\prime },e_{2}\right)$ which contradicts the inductive hypothesis that $d\left(T_{1}^{\prime },T_{2}^{\prime }\right)=m_{1}-1+m_{2}-1$.

A special case of the theorem is when both trees *T*_1_ and *T*_2_ are binary trees, i.e., *T*_1_, $T_{2}\in T\left(n,n-3\right)$. In this case there exist {1, 2, …, *n*}-labelings of the leaves such that *d*(*T*_1_, *T*_2_) = 2*n* − 6 for *n* ≥ 3.

For unrooted trees *T*_1_ and *T*_2_ that are not necessarily proper, i.e., where it is possible that internal nodes have degree 2, Theorem 1 implies the following corollary.

**Corollary 1.** *For each two unrooted trees* $T_{i}\in T\left(n,m_{i}\right)$, *n* ≥ 3, *i* ∈ {1, 2} *where* *k*_*i*_ *is the number of internal nodes with degree 2 in* *T*_*i*_ *there exist* {1, 2, …, *n*}-*labelings of the leaves of both trees such that for the corresponding phylogenetic trees* *d*(*T*_1_, *T*_2_) = *m*_1_ − *k*_1_ + *m*_2_ − *k*_2_.

*Proof*. To see that the corollary holds consider the case that in one of the trees *T*_*i*_ there exists a path of maximal length with internal nodes *n*_1_, *n*_2_, …, *n*_*j*_ that all have degree 2. Then, there exist nodes *n*_0_ and *n*_*k*+1_ not in the path such that *n*_0_ is connected to *n*_1_ and *n*_*k*_ is connected *n*_*k*+1_. For each two edges *e*, *e*′ that are incident to (at least) one of the nodes in the path *π*(*T*, *e*) = *π*(*T*, *e*′) holds. Hence, if the path *n*_1_, *n*_2_, …, *n*_*j*_ is removed from *T*_*i*_ and exchanged by a single edge (i.e., *n*_0_ is connected to *n*_*k*+1_) for the resulting tree *T*′ the equality *P*(*T*′) = *P*(*T*) holds. Iteratively, apply this procedure until all *k*_1_ + *k*_2_ internal nodes with degree 2 have been removed in both trees and apply Theorem 1 to the resulting trees.

For rooted topological trees we show in the following a theorem that is analogous to Theorem 1 for unrooted trees and gives a bound on the cluster distance of two corresponding phylogentic trees.

**Theorem 2.** *For each two proper rooted topological trees* $T_{1}\in T_{r}\left(n,m_{1}\right)$ *and* $T_{2}\in T_{r}\left(n,m_{2}\right)$ *there exist* {1, 2, …, *n*}-*labelings of the leaves of both trees such that* *d*_*r*_(*T*_1_, *T*_2_) = *m*_1_ + *m*_2_ *with* *n* ≥ 3, 0 ≤ *m*_1_ ≤ *n* − 2, 0 ≤ *m*_2_ ≤ *n* − 2.

*Proof*. The proof is similar to the proof of Theorem 1. We show that there exist labelings for the leaves of *T*_1_ and *T*_2_ such that for each internal node *v*_1_ of *T*_1_ the cluster *T*_1_(*v*_1_) is not contained in *Cl*(*T*_2_) and for each internal node *v*_2_ of *T*_2_ the cluster *T*_2_(*v*_2_) is not contained in *P*(*T*_1_). The proof is done by induction on *n*.

Base case *n* = 3. In this case each tree has either no internal node (then all three leaves are connected to the root) or it has one internal node that is connected to two leaves and the other leaf is connected to the root. In the first case a tree has only trivial clusters and in the second case it has exactly one non-trivial cluster that contains the labels of the two leaves that are connected to the internal node. If the second case holds for both trees then the two leaves that are connected to the internal node can get labels 1 and 2 for *T*_1_ respectively labels 1 and 3 for *T*_2_ and the theorem holds. Otherwise, the theorem holds for all {1, 2, 3}-labelings of *T*_1_ and *T*_2_.

For the inductive step consider two trees *T*_1_ and *T*_2_ with *n* ≥ 4 leaves and the following three cases:

1. One of the trees has no internal node.
2. Both trees have (at least) one internal node that is connected to at least 3 leaves.
3. Both trees have (at least) one internal node and for (at least) one of the trees each internal node is connected to at most two leaves.

Proof for case (1). W.l.o.g. let *T*_1_ be a tree that has no internal node and therefore *Cl*(*T*_1_) contains only trivial clusters. Then, for each labeling of the leaves of *T*_2_ and any internal node *v* of *T*_2_ a non-trivial *cl*(*T*_2_, *v*) is not in *Cl*(*T*_1_) and the theorem holds.

Proof for case (2). Let *u* (*x*) be an internal node in *T*_1_ (respectively *T*_2_) that is connected to at least 3 leaves. From each tree remove one of the leaves connected to *u*, respectively *x*. The resulting trees $T_{1}^{\prime }$ and $T_{2}^{\prime }$ are both proper and have *n* − 1 leaves and *m*_1_ (respectively *m*_2_) internal nodes. By the induction hypothesis there exist {1, 2, …, *n* − 1}-labelings for $T_{1}^{\prime }$ and $T_{2}^{\prime }$ such that $d_{r}\left(T_{1}^{\prime },T_{2}^{\prime }\right)=m_{1}+m_{2}$. Extend these labelings to {1, 2, …, *n*}-labelings for *T*_1_ and *T*_2_ by assigning the label *n* to both removed leaves. Since for each two internal nodes *v* of $T_{1}^{\prime }$ and *y* of $T_{2}^{\prime }$ the clusters $cl(T_{1}^{\prime },v)$ and $cl(T_{2}^{\prime },y)$ are different the clusters *cl*(*T*_1_, *v*) and *cl*(*T*_2_, *y*) are also different. Hence, *d*_*r*_(*T*_1_, *T*_2_) = *m*_1_ + *m*_2_

It remains to prove case (3). W.l.o.g. assume that in *T*_1_ each internal node is connected to at most two leaves. Since *T*_1_ is a proper tree there exists an internal node *u* of *T*_1_ that is neighbour to exactly two leaves *u*_1_ and *u*_2_ and has exactly one other neighbour *v* (which is an internal node or the root). Consider the tree $T_{1}^{\prime }$ that is obtained from *T*_1_ by removing nodes *u*_1_, *u* and edges {*u*_1_, *u*}, {*u*, *v*} and by connecting node *u*_2_ to *v*. Then *T*_1_ is proper and in $T_{r}\left(n-1,m_{1}-1\right)$.

First assume that *T*_2_ has a node *x* that is connected to at least three leaves *x*_1_, *x*_2_, and *x*_3_. Construct $T_{2}^{\prime }$ by removing leaf *x*_1_. Then, $T_{2}^{\prime }$ is proper and in $T_{r}\left(n-1,m_{2}\right)$. By the induction hypothesis there exist {1, 2, …, *n* − 1}-labelings for $T_{1}^{\prime }$ and $T_{2}^{\prime }$ such that $d_{r}\left(T_{1}^{\prime },T_{2}\right)=m_{1}-1+m_{2}$. Extend these labelings to {1, 2, …, *n*}-labelings for *T*_1_ and *T*_2_ by assigning leaves *u*_1_ and *x*_1_ the label *n*. Clearly, no cluster of *Cl*(*T*_2_) is equal to a cluster *cl*(*T*_1_, *w*) where *w* is an internal node with *w* ≠ *u*. Also, *cl*(*T*_1_, *u*) = {*n*, *i*} for an *i* ∈ [1, *n* − 1] is not in *Cl*(*T*_2_) because all cluster in *Cl*(*T*_2_) that include *n* have at least three elements. Thus, *d*(*T*_1_, *T*_2_) = *m*_1_ + *m*_2_ holds.

It remains to consider the case that all internal nodes in *T*_2_ are connected to at most two leaves. Then there must exist an internal node *x* in *T*_2_ that is connected to two leaves *x*_1_ and *x*_2_ and to exactly one other node *y* that is an internal node or the root. Similar as for *T*_1_, create a phylogenetic tree $T_{2}^{\prime }$ by removing nodes *x*_1_, *x* and edges {*x*_1_, *x*}, {*x*, *y*} from *T*_2_ and by connecting *x*_2_ to *y*. By construction, $T_{2}^{\prime }$ is proper and in $T_{r}\left(n-1,m_{2}-1\right)$. By the induction hypothesis there exist {1, 2, …, *n* − 1}-labelings for $T_{1}^{\prime }$ and $T_{2}^{\prime }$ such that *d*_*r*_(*T*_1_, *T*_2_) = *m*_1_ − 1 + *m*_2_ − 1. Now consider four subcases.

Case a: Both nodes *v* and *y* have at least 2 neighbouring leaves in $T_{1}^{\prime }$ respectively $T_{2}^{\prime }$. Then, we can assume w.l.o.g. that leaf *u*_2_ has label *i* in $T_{1}^{\prime }$ and leaf *x*_2_ has label *j* in $T_{2}^{\prime }$ with *j* ≠ *i* (Because, otherwise, the label of *u*_2_ can be exchanged with the label of another leaf that is connected to *v*). Now, extend the labelings to {1, 2, …, *n*}-labelings for *T*_1_ and *T*_2_ by assigning both leaves *u*_1_ and *x*_1_ label *n*. Clearly, cluster *cl*(*T*_1_, *u*) is not in *Cl*(*T*_2_) and cluster *cl*(*T*_2_, *x*)) is not in *Cl*(*T*_1_). Hence, *d*_*r*_(*T*_1_, *T*_2_) = *m*_1_ + *m*_2_ easily follows.

Case b: Node *v* has at least two neighbouring leaves (one leaf is *u*_2_ and let *v*_1_ be the other leaf) in $T_{1}^{\prime }$ and node *y* has only one neighbouring leaf (i.e., leaf *x*_2_) in $T_{2}^{\prime }$. Assume that *j* is the label of *x*_2_. Then at least one *u*_2_ and *v*_1_ has a label *i* ≠ *j*. Assume first, that node *u*_2_ has label *i* and extend the labelings of $T_{1}^{\prime }$ and $T_{2}^{\prime }$ to {1, 2, …, *n*}-labelings for *T*_1_ and *T*_2_ by assigning label *n* to leaves *u*_1_ and *x*_1_. Then the cluster *cl*(*T*_1_, *u*) is not in *Cl*(*T*_2_) and the cluster *cl*(*T*_2_, *x*) is not in *Cl*(*T*_1_). It follows easily that *d*_*r*_(*T*_1_, *T*_2_) = *m*_1_ + *m*_2_ holds. It remains to consider the case that node *v*_1_ has label *i*. Then exchange the labels of *u*_2_ and *v*_1_ in $T_{1}^{\prime }$. Clearly, for this labeling $d_{r}\left(T_{1}^{\prime },T_{2}^{\prime }\right)=m_{1}-1+m_{2}-1$ holds. Now, proceed as before to show the result.

Case c: Node *v* has only one neighbouring leaf in $T_{1}^{\prime }$ and node *y* has at least 2 neighbouring leaves in $T_{2}^{\prime }$. This case is symmetric to Case (b) and the proof is analogously.

Case d: Nodes *v* and *y* have only one neighbouring leaf in $T_{1}^{\prime }$ respectively $T_{2}^{\prime }$. For the chosen {1, 2, …, *n* − 1}-labelings for $T_{1}^{\prime }$ and $T_{2}^{\prime }$ with $d_{r}\left(T_{1}^{\prime },T_{2}^{\prime }\right)=m_{1}-1+m_{2}-1$ let *i* and *j* be the labels of *u*_2_ respectively *x*_2_. If *i* ≠ *j* extend the labelings to {1, 2, …, *n*}-labelings for *T*_1_ and *T*_2_ by assigning label *n* to leaves *u*_1_ and *x*_1_. Then, cluster *cl*(*T*_1_, *u*) is not in *Cl*(*T*_2_) and cluster *cl*(*T*_2_, *x*) is not in *Cl*(*T*_1_). Now, it is not hard to show that *d*_*r*_(*T*_1_, *T*_2_) = *m*_1_ + *m*_2_ holds.

It remains to consider the case *i* = *j*. Let *k* ∈ {1, 2, …, *n* − 1} be a label with *k* ≠ *i*. First, extend the labelings of $T_{1}^{\prime }$ and $T_{2}^{\prime }$ to {1, 2, …, *n*}-labelings for *T*_1_ and *T*_2_ by assigning label *n* to leaves *u*_1_ and *x*_1_. Then change the labeling for *T*_1_ by exchanging the labels of the leaves with labels *k* and *n* and for $T_{1}^{\prime }$ by exchanging the label of the leave with label *k* by *n*. Now, cluster *cl*(*T*_1_, *u*) = {*k*, *i*} is not in *Cl*(*T*_2_). Similarly, cluster *cl*(*T*_2_, *x*) = {*n*, *i*} is not in *Cl*(*T*_1_). By the construction it holds for each cluster in *Cl*(*T*_1_) that it contains either *i* and *k* or none of them. Similarly, it holds for each cluster in *Cl*(*T*_2_) that it contains either *i* and *n* or none of them. Hence, for each internal node *w* ≠ *u* in *T*_1_ it holds: either *i* ∉ *cl*(*T*_1_, *w*) and $cl\left(T_{1},w\right)=cl\left(T_{1}^{\prime },w\right)$ or *i*, *k* ∈ *cl*(*T*_1_, *w*) and $cl\left(T_{1},w\right)-\left\{k\right\}=cl\left(T_{1}^{\prime },w\right)$. Similarly, for each internal node *z* ≠ *x* in *T*_2_ it holds: either *i* ∉ *cl*(*T*_2_, *z*) and $cl\left(T_{2},z\right)=cl\left(T_{2}^{\prime },z\right)$ or *i*, *n* ∈ *cl*(*T*_2_, *z*) and $cl\left(T_{2},z\right)-\left\{n\right\}=cl\left(T_{2}^{\prime },z\right)$.

Now, it remains to show that for each *cl* ∈ *Cl*(*T*_1_), *cl* ≠ {*k*, *i*} implies *cl* ∉ *Cl*(*T*_2_) and, vice versa, for each *cl* ∈ *Cl*(*T*_2_), *cl* ≠ {*n*, *i*} implies *cl* ∉ *Cl*(*T*_1_). To show the first statement, let *cl* ∈ *Cl*(*T*_1_), *cl* ≠ {*k*, *i*}. There exist four cases:

- Case i) *i*, *n* ∉ *cl*. Then *k* ∉ *cl* and $cl\in Cl(T_{1}^{\prime })$. Therefore, $cl\;\notin \;Cl(T_{2}^{\prime })$. By the construction it follows that *cl* ∉ *Cl*(*T*_2_).
- Case ii) *i* ∉ *cl*, *n* ∈ *cl*. Then *cl* ∉ *Cl*(*T*_2_) because every cluster in *Cl*(*T*_2_) contains either *i* and *n* or none of them.
- Case iii) *i* ∈ *cl*, *n* ∉ *cl*. Similar as in case (ii) it follows that *cl* ∉ *Cl*(*T*_2_).
- Case iv) *i*, *n* ∈ *cl*. Then *k* ∈ *cl* and $cl-\left\{k\right\}\in Cl\left(T_{1}^{\prime }\right)$. Therefore, $cl-\left\{n\right\}\;\notin \;Cl\left(T_{2}^{\prime }\right)$. By the construction it follows that *cl* ∉ *Cl*(*T*_2_).

The second statement, i.e., *cl* ∈ *Cl*(*T*_2_), *cl* ≠ {*n*, *i*} implies *cl* ∉ *Cl*(*T*_1_), can be shown by symmetric arguments. Thus, the theorem holds.

Similarly, as for unrooted trees the following corollary can be shown for rooted trees *T*_1_ and *T*_2_ that are not necessarily proper, i.e., where it is possible that internal nodes have degree 2. Theorem 2 implies the following corollary.

**Corollary 2.** *For each two rooted trees* $T_{i}\in T_{r}\left(n,m_{i}\right)$, *n* ≥ 3, *i* ∈ {1, 2} *where* *k*_*i*_ *is the number of internal nodes with degree 2 in* *T*_*i*_ *there exist* {1, 2, …, *n*}-*labelings of the leaves of both trees such that for the corresponding phylogenetic trees* *d*_*r*_(*T*_1_, *T*_2_) = *m*_1_ − *k*_1_ + *m*_2_ − *k*_2_.

*Proof*. To see that the corollary holds consider the case that in one of the trees *T*_*i*_ exists a path *n*_1_, *n*_2_, …, *n*_*k*_ of maximal length such that all nodes *n*_*j*_ of the path have degree 2. Let *n*_*k*_ be the node of the path that is farthest away from the root and let *n*_*k*+1_ be a node that is connected with *n*_*k*_ and is not on th path. Clearly such a node *n*_*k*+1_ must exist and *n*_*k*+1_ is a leaf or an internal node with degree ≥ 3. Let *n*_0_ be a node that is connected to *n*_1_ and is not on the path. Clearly such a node *n*_0_ must exist and *n*_0_ is the root or an internal node with degree ≥ 3. For each two node *n*_*j*_, 1 ≤ *j* ≤ *k* holds *cl*(*T*, *n*_*j*_) = *cl*(*T*, *n*_*k*+1_). Hence, if the path *n*_1_, *n*_2_, …, *n*_*k*_ is removed from *T*_*i*_ and exchanged by a single edge that connects *n*_0_ and *n*_*k*+1_ for the resulting tree $T_{i}^{\prime }$ the equality $Cl\left(T_{i}\right)=Cl\left(T_{i}^{\prime }\right)$ holds. Iteratively, apply this procedure until all *k*_1_ + *k*_2_ internal nodes with degree 2 have been removed from both trees and apply Theorem 1 to the resulting trees.

From Corollary 2 we obtain the following corollary on the worst case of the unrooted CD distance between an unrooted phylogenetic tree and a roooted phylogenetic tree both with *n* leaves.

**Corollary 3.** *For each unrooted tree* $T_{1}\in T\left(n,m_{1}\right)$ *and rooted tree* $T_{2}\in T_{r}\left(n,m_{2}\right)$, *n* ≥ 3 *where* *k*_*i*_ *is the number of internal nodes with degree 2 in* *T*_*i*_, *i* ∈ 1, 2 there exist {1, 2, …, *n*}-*labelings of the leaves of both trees such that for the corresponding phylogenetic trees* *d*_*ur*_(*T*_1_, *T*_2_) = *m*_1_ − *k*_1_ + *m*_2_ − *k*_2_.

*Proof*. For an edge *e* of *T*_1_ consider the rooted tree *T*_1,*e*_. By definition of *T*_1,*e*_ it holds that $T_{1,e}\in T_{r}\left(n,m_{1}\right)$ and *T*_1,*e*_ has *k*_1_ internal node that have degree 2. To see this, recall that the root is not an inner node. Now, the corollary follows immediately from Corollary 2.

Note, that the proof of Corollary 3 has shown that for any edge *e* of *T*_1_ is holds that there exists {1, 2, …, *n*}-labelings of the leaves of trees *T*_1,*e*_ and *T*_2_ such that *d*_*r*_(*T*_1,*e*_, *T*_2_) = *m*_1_ − *k*_1_ + *m*_2_ − *k*_2_.

## Conclusion

It was shown that for two topological trees *T*_1_ and *T*_2_ with *n* leaves, *m*_*i*_ internal edges in tree *T*_*i*_, and *k*_*i*_ nodes of degree 2 in *T*_*i*_, *i* ∈ 1, 2 there exists assignments of labels {1, 2, …, *n*} to the leaves of each tree such that the tree partition distance (TPD; also called Robinson-Foulds distance for unrooted trees) between the corresponding unrooted phylogenetic trees is *m*_1_ − *k*_1_ + *m*_2_ − *k*_2_. In addition, this number is an upper bound, i.e., there does not exist assignments of labels {1, 2, …, *n*} to the leaves such that the TPD between both trees is larger than *m*_1_ − *k*_1_ + *m*_2_ − *k*_2_. Moreover, it was shown that analogous results hold for the cluster distance (CD; also called Robinson-Foulds distance for rooted trees) of two rooted trees and for the unrooted cluster distance (urCD) of an unrooted tree and a rooted tree. Our results can be used to compute a normalized version of the corresponding distance measures.
